# Three discipline collaborative radiation therapy (3DCRT) special debate: A physicist's time is better spent in direct patient/provider interaction than in the patient's chart

**DOI:** 10.1002/acm2.13559

**Published:** 2022-02-15

**Authors:** Todd F. Atwood, Narottam Lamichhane, Krisha Howell, Stephanie E. Weiss, Louise Bird, Charles Pearson, Michael C. Joiner, Michael M. Dominello, Jay Burmeister

**Affiliations:** ^1^ Department of Radiation Medicine and Applied Sciences University of California San Diego California USA; ^2^ Department of Radiation Oncology University of Maryland Baltimore Maryland USA; ^3^ Department of Radiation Oncology Fox Chase Cancer Center Philadelphia Pennsylvania USA; ^4^ Saskatchewan Cancer Agency Regina Canada; ^5^ Charles Pearson Associates, Inc Bellevue Washington USA; ^6^ Department of Oncology Wayne State University School of Medicine Detroit Michigan USA; ^7^ Gershenson Radiation Oncology Center Barbara Ann Karmanos Cancer Institute Detroit Michigan USA

**Keywords:** patient communication, patient interaction

## 1 THREE DISCIPLINE COLLABORATIVE RADIATION THERAPY (3DCRT) DEBATE SERIES

Radiation oncology is a highly multidisciplinary medical specialty, drawing significantly from three scientific disciplines—medicine, physics, and biology. As a result, discussion of controversies or changes in practice within radiation oncology involves input from all three disciplines. For this reason, significant effort has been expended recently to foster collaborative multidisciplinary research in radiation oncology, with substantial demonstrated benefit.^1^, ^2^ In light of these results, we have adopted this “team‐science” approach to the traditional debates featured in this journal. This article is part of a series of special debates entitled “three discipline collaborative radiation therapy (3DCRT)”, in which each debate team has included three multidisciplinary team members, with the hope that this format would be both engaging for the readership and foster further collaboration in the science and clinical practice of radiation oncology. All 3DCRT debates thus far have included a radiation oncologist, medical physicist, and radiobiologist on each team. For this debate, we break that trend and include a patient representative along with a radiation oncologist and medical physicist on each team. We hope this patient perspective adds a valuable new aspect to our debate format and encourages the continued inclusion of patient perspectives in future clinical discussions.

## 2 INTRODUCTION

Medical physicists have historically contributed to patient care in radiation oncology primarily through the implementation and oversight of technology and comprehensive quality and safety programs.^3^, ^4^ However, with the introduction of more robust equipment and widespread automation, we anticipate that the time required for these tasks will decrease accordingly.^5^, ^6^ In addition, our dynamic healthcare environment continuously pressures the medical profession to redefine its contribution and value. So where can a more unfettered medical physicist provide “top of the license” contributions to the quality of patient care? One recent effort has been to cultivate increased engagement of the medical physicist with the patient for the intended result of maximizing the patient's understanding of their treatment and improving the overall healthcare experience. But does the implementation of a direct, patient‐facing role for the physicist result in a substantial improvement in the patient experience and/or the quality of care? And if so, does this outweigh the value of other possible technical contributions to which physicists could re‐allocate their time? Quality and safety initiatives will presumably grow as health care organizations and accreditors work to implement meaningful patient safety programs with defined executive responsibilities and accountability to specific outcomes. Does direct patient care by the physicist take time away from the physicist's contributions to these quality and safety initiatives, or is this patient interaction a valuable and critical component of such initiatives? In other words, where does the medical physicist most enhance the quality of care in radiation oncology—with the patient's technology, or with the patient? This is the subject of this month's three discipline collaborative radiation therapy debate.

Arguing for the proposition will be Dr. Todd Atwood, Dr. Krisha Howell, and Mr. Charles Pearson. Dr. Atwood is an Associate Professor and Senior Associate Division Director of Transformational Clinical Physics at UC San Diego. As a native of North Carolina, Todd attended the University of North Carolina at Chapel Hill, before receiving his MS and PhD degrees from Wake Forest University. After completing a medical physics residency at Stanford University, he began to focus on maximizing the impact medical physicists have on patient care. Dr. Howell is currently an Associate Professor in Gynecologic and Sarcoma Radiation Oncology and acting Clinical Director at Fox Chase Cancer Center/Temple University. Originally from Southeastern Michigan, she received her Medical Doctorate at Wayne State University School of Medicine. She completed a residency in Radiation Oncology at the Medical University of Charleston. Additionally, she received brachytherapy fellowship training at Princess Margaret Hospital. Charles Pearson is the patient partner for the proposition. Mr. Pearson received his B.A. degree in economics from Seattle University and his M.P.H. degree in hospital administration from UC Berkeley. Prior to his management consulting career, he spent 7 years in line executive positions in both academic and non‐academic (300–500+ bed) acute care hospitals. During his 40 years of directly providing management consulting services to hospitals and healthcare systems, he contracted with approximately 200 clients in 39 states.

Arguing against the proposition will be Dr. Narottam Lamichhane, Dr. Stephanie Weiss, and Ms. Louise Bird. Dr. Lamichhane is an assistant professor and medical physicist in the Department of Radiation Oncology at the University of Maryland School of Medicine. He completed his therapeutic medical physics residency from the University of Miami Miller School of Medicine. His training and research interests focus on treatment planning, quality assurance, imaging, and experimental therapeutics. Dr. Weiss is a professor in the Department of Radiation Oncology, Chief of the Division of Neurologic Oncology, and Director of the Radiation Oncology Residency and Fellowship Training Program at Fox Chase Cancer Center/Temple University. She completed her residency training at Johns Hopkins Hospital and has also served as an attending physician for Brigham and Women's Hospital/Dana Farber Cancer Institute and on the faculty at Harvard Medical School. Louise Bird is the patient partner against the proposition. She lives and works in rural Saskatchewan, Canada, and is a breast cancer survivor of 18 years. She participates in many different Provincial and Pan Canadian initiatives, including serving as co‐chair of the Patient and Family Advisory Council for the Saskatchewan Cancer Agency. For more about her patient story, see https://cancerfoundationsask.ca/patient/louise‐bird/.

## 3 OPENING STATEMENTS

### 3.1 Todd Atwood, PhD; Krisha Howell, MD; Charles Pearson, MPH

As the field of radiation oncology has evolved, so has the role of the medical physicist. While the primary function of the medical physicist in radiation oncology has always centered around the design and delivery of safe and efficacious therapy, the day‐to‐day responsibilities of medical physicists have consistently adapted to provide patients with the highest level of care. To assure the continued value of the medical physicist in the changing healthcare landscape, the American Association of Physicists in Medicine (AAPM) created a new initiative, called “Medical Physics 3.0” (https://www.aapm.org/MedPhys30/), which aims to “redefine and reinvigorate the role of physics in modern medicine.”^7^

When evaluating the current needs of radiation oncology patients, one desire stands out—patients want to be more involved in their care. Research has shown that radiation oncology patients want comprehensive and detailed information about their disease and treatment procedures^8^; however, this is not always easily achieved. From the perspective of the patient, radiation oncology is often viewed as a complex and overwhelming medical specialty. After receiving a cancer diagnosis, patients are quickly introduced to an array of complicated imaging and treatment modalities, often with little understanding of the role they play in their care. In addition, patients also frequently face concerns and misconceptions about the use of radiation and how it can safely and effectively treat their disease. These circumstances commonly result in patients looking for answers and information about the technical aspects of their care online, where even the most reliable sources have been shown to be nonspecific or too complicated for the general public.^9^, ^10^ Unfortunately, all of these factors have the potential to negatively influence the patient experience. More importantly, the combination of these factors can create anxiety and patient‐related distress, which has been shown to negatively influence outcomes following radiation therapy.^11^

Ensuring that all patients have the information they need to understand and feel comfortable with their care is a necessity for the field of radiation oncology. Medical physicists are ideally positioned to help address some of these concerns by leading efforts to demystify the radiation therapy process for patients. Using their comprehensive knowledge of the technology involved in radiation oncology and the specifics of each patient's treatment plan, medical physicists could ensure that all patient questions and concerns related to the technical aspects of their care are adequately addressed. Additionally, research has shown that education assists with patient enlistment in their own care, which can lead to improved adherence to treatment regimens.^12^

Traditionally, medical physicists have had some patient contact, but these interactions have typically been limited to brief clinical encounters or meetings with technologically savvy and inquisitive patients. Recently, more comprehensive patient‐facing roles have been explored to evaluate the potential of further integrating medical physicists into direct patient care. One example is including the medical physicist in the initial radiation oncology consult to facilitate a collaborative approach to patient care at the beginning of treatment.^13^ This process introduces the patient to both the medical and technical experts on the care team and creates an opportunity for the radiation oncologist and medical physicist to transfer knowledge at an early stage in the treatment planning process.

More extensive direct patient interactions by the medical physicist have also been studied. As part of the Physics Direct Patient Care protocol, medical physicists established independent professional relationships with patients to oversee and communicate all of the technical aspects related to the patient's care.^14^ After attending a dedicated patient communication training program, medical physicists routinely met with patients for two physicist–patient consults to describe the role of a medical physicist, explain the treatment planning and delivery process, review the patient's treatment plan, and answer all technical questions. The results from this trial indicated that physicist–patient consults were associated with statistically significant decreases in patient anxiety and increases in patient satisfaction.

In addition to improving the patient experience, patient‐facing roles for medical physicists would also strengthen clinical collaborations with radiation oncologists. Effective communication and teamwork have traditionally been assumed to be skills of expert individual practitioners, and formal training and assessment in these areas has been largely absent. By expanding the direct patient care team to include medical physicists, opportunities for shared decision‐making would arise and communications bridging the technical and medical aspects of patient care would increase. This approach works to create a well‐understood plan of care, which greatly reduces the chances of errors becoming consequential and injuring patients, and expresses a culture of strong, clear, and visible attention to safety.^15^, ^16^

### 3.2 Narottam Lamichhane, PhD; Stephanie Weiss, MD; Louise Bird

The field of radiation oncology is interdisciplinary and requires a lot of teamwork. In the midst of this teamwork, the physicist plays a vital role in maintaining patient safety and quality of care. This delicate balance of teamwork in radiation oncology requires each division to prioritize and focus on their expertise. The smooth workflow of the radiation therapy department is facilitated by each team member carrying out their required work with diligence. A safety gate of this entire workflow is the division of physics, and a major focus of routine radiation oncology physics work is chart review. The process of chart review occurs within various steps of a clinical physics workflow such as pretreatment initial chart review, weekly chart review, and end of treatment chart review. The initial chart review is one of the most effective ways of diagnosing pretreatment errors and ensuring compliance with the prescription.^17^ Since the largest number of errors occurs during the planning and the pretreatment processes, chart review represents an opportunity to improve the quality assurance of the entire workflow.^18^ Similarly, the weekly chart review also plays a significant role in providing quality control during the course of patient treatment to catch or rule out any gross errors. In the current state of the radiation therapy workflow, the treating radiation oncologist, nurse, and clinical care team perform the direct patient interaction.

The motivation behind the physicist being involved in direct patient care is noteworthy. The responsibilities of clinical physicists are evolving in the current era. However, adding direct patient care as another responsibility of a clinical physicist also comes with many challenges. For a radiation oncology department, and specifically for the division of physics, the allotment of staff is based on various factors within the department and guidance from professional societies. As such, the number of physicists required for a radiation therapy department is guided by the number of treatments, radiation oncologists, machines, special procedures, and many other clinical factors. The addition of direct patient interaction will add extra responsibilities to the established physics workflow that may not only detract from completing existing responsibilities, but also lead to miscalculation in terms of allocation of medical physicist FTE in a given department, hospital, or hospital system. As such, this will require restructuring the standard physics workflow within the department and may require additional physics resources that may not always be feasible. Most importantly, and realistically, additional responsibilities without additional staffing would likely result in a reduction in the amount of time available for traditional physics work such as chart review. This may cause a strain on the physics team that leads to potential errors and the compromise of patient safety.

The American Society for Radiation Oncology published details surrounding implementation of best clinical practices in its “safety is no accident” report, and emphasizes the open communication between different divisions within the department.^19^ In our interpretation, clear communication between physician and physicist requires effective information sharing without impinging on the predefined responsibilities of an individual division. This allows physicists to be involved in the clinical decision‐making and still focus on the patient safety that will ultimately ensure the highest quality clinical care for patients.

Furthermore, the involvement of the physicist in patient care does not necessarily have to involve direct interaction. In light of the current common telehealth practices, the introduction of the care team with live video or a recorded video may provide the patient the assurance they need. Additionally, during the development of a patient's individualized treatment, the information on various treatment techniques as well as the physics behind the treatment modalities and peer review process can be shared with patients either through brochures or as a recorded video, thus assuring patients that safe and effective protocols are being practiced. Finally, a detailed discharge summary, drafted and reviewed by the care team, will be provided to the patient and to their primary care provider, which provides information about the radiotherapy treatment.

## 4 REBUTTAL

### 4.1 Todd Atwood, PhD; Krisha Howell, MD; Charles Pearson, MPH

Our colleagues argue that expanding the role of the medical physicist would detract from quality and safety and, moreover, contradict radiation oncology staffing models. Although we respect their concern for patient safety, we believe it is misplaced. More engaged patient‐facing roles and responsibilities would strengthen quality and safety efforts, while optimizing patient care and education by leveraging the medical physicist's unique strengths.

As our colleagues mentioned, chart review is an integral, but time consuming, part of a quality and safety program. However, the time required for these tasks is decreasing as software improves and automated tools for clinical decision support are introduced.^5^, ^20^ As this trend continues, we anticipate medical physicists will have the freedom to break from rote tasks and better populate their workload with “top of license” activities. We see these activities defined as cultivating direct patient care roles to educate patients on the technical aspects of their treatment, to enlist patients in their own care, and to further engage in the technical decisions of a patient's own treatment plan design. These roles could lead to improved treatment adherence,^12^ result in shared technical decision making with the radiation oncologist, and foster increased communication among experts on the care team. All of these factors have the potential to bring about dramatic improvements in quality and safety.^16^

Furthermore, we challenge our colleagues’ concerns for medical physicist involvement in “patient care” as a misconception. The physicist as an active participant in patient care is securely set within the current purview of the medical physicist. Our colleagues’ misgiving may result from, as it stands, the lack of public transparency to the physicist's role in that care. We wish to increase this transparency and reinforce that, in the radiation treatment paradigm, highest quality care of the patient is not just the responsibility of the physician, but also an outcome for which the physicist shares accountability.

The value a medical physicist has in patient care is evident in expectations built into the current patient relations and clinic flow. It, however, is not often executed in a manner to build the patient's education and trust. Yet, these elements are highly crucial. In a review of more than 8,000 patient satisfaction surveys, albeit missing a medical physicist component question, patient satisfaction was greatest with regard to their perceived provider relationships.^21^ Beyond that, there exists a uniqueness to the relationship between a medical physicist and patient, some of which cannot be supplemented. We need to look no further than the standard procedure for HDR remote afterloader major medical emergencies. As per safety protocol, a solo physicist initially enters the treatment room in a timely manner to attempt retraction of the source.^22^ In such an emergency scenario, it is highly plausible that the physicist and patient would occupy the same physical space alone in a high‐pressure, time‐sensitive moment. If the patient was not familiar with whom this individual was or what tasks they are authorized to perform, it could add anxiety, increase time, and decrease patient cooperation while attempting to extract the source to limit radiation exposure.

To summarize, although direct patient interactions represent a new role for medical physicists, the evolution of the profession should be driven by the needs of radiation oncology patients, not confined by historical responsibilities. Current data suggests patients want extensive information about their disease and treatment procedures^8^ and that physicist‐patient consults have the ability to address these needs, with low patient anxiety and high patient satisfaction.^14^ In addition, recent studies have shown that a comprehensive patient communication training program for medical physicists can be created by tailoring the accepted medical school curriculum,^23^ and that this type of program has the ability to increase the participants’ level of confidence across multiple communication categories.^24^

More broadly, the development of direct patient care roles for medical physicists has the potential to facilitate professional growth within the field of radiation oncology as a whole. As medical physicists take on new responsibilities, radiation oncologists will have more time for clinical tasks, such as multi‐disciplinary clinics and tumor boards, which will help cultivate larger roles in oncologic management for all cancer patients.^25^, ^26^

### 4.2 Narottam Lamichhane, PhD; Stephanie Weiss, MD; Louise Bird

We agree with the opposing team that the scope of medical physicists has evolved over the years. We also agree that re‐fitting the vocation within the ever‐changing dynamics of radiation oncology is the right approach for medical physicists. However, modern needs are not best served by direct patient care by medical physicists. Indeed, medicine has never involved patient interaction by all players. Clinically trained pathologists and radiologists best serve patients solely through direct peer‐to‐peer interaction. So too do medical physicists. The collaborative division of expertise in a functioning department of radiation oncology is akin to the clinical division of expertise that is enhanced by bringing these experts together in multi‐disciplinary conferences. This promotes seamless throughput of patient care without compromising patient safety. We agree that patient awareness of the type of treatment and the methodology of treatment they are receiving is of utmost importance. However, this is not necessarily best achieved with direct physicist–patient interaction. Patient‐related information sharing can be accomplished by electronic means, or printed materials provided by the patient's established clinical care team. This method is not only more efficient and cost‐effective but potentially less overwhelming for the patient.

We agree with the opposing team that medical physicists should use their technological expertise to increase the visibility of medical physics. However, providing each patient with a consultation by a physicist will require significant departmental resources, add an unnecessary burden to the patient in terms of increased time with the ever‐expanding team, and may even depersonalize the experience. Is it not possible that the physician and the physicist may articulate the different rationale for particular recommendations or details of a treatment plan? Any discrepancy or perceived discrepancy has the potential to add stress and cause additional anxiety. Finally, even if the benefits were agreed to outweigh the risks (which we do not at this time concede), not every radiation oncology department will have sufficient resources to accommodate such costly time commitment from the physicist. Therefore, the inclusion of direct patient care duties must be evaluated with a view of the cost–benefit analysis impact on the medical physicist and the radiation oncology department.^13^

The opposing team brings up a good point regarding the new initiative by AAPM called “MedPhys 3.0” (https://www.aapm.org/medphys30/), and we support this initiative to redefine and reinvigorate the practice of medical physics. One of the initiatives of MedPhys 3.0 is to promote new physics contributions in all areas of medicine including domains beyond radiation medicine. The foundation of medicine is its underlying research. Medical physics research plays an important role in shaping the field of radiation oncology. Thus, we believe that the MedPhys 3.0 initiative may be best achieved by extending medical physics research into contemporary fields of medicine in lieu of directing physics efforts in direct patient interaction.

## AUTHOR CONTRIBUTIONS

All authors were responsible for preparation of arguments, and writing and reviewing the manuscript.

## CONFLICT OF INTEREST

The authors declare no conflict of interest.
